# Retrospective analysis of efficacy and safety of recombinant human Rh-endostatinstatin combined with concurrent radiotherapy for cervical cancer

**DOI:** 10.12669/pjms.40.8.8770

**Published:** 2024-09

**Authors:** Xin Zhang, Qian Li, Kuan Liu, Hong-yun Shi

**Affiliations:** 1 Xin Zhang, Department of Radiotherapy, Affiliated Hospital of Hebei University, Baoding 071000, Hebei, China; 2 Qian Li, Department of Radiotherapy, Affiliated Hospital of Hebei University, Baoding 071000, Hebei, China; 3 Kuan Liu, Department of Radiotherapy, Affiliated Hospital of Hebei University, Baoding 071000, Hebei, China; 4 Hong-yun Shi, Department of Radiotherapy, Affiliated Hospital of Hebei University, Baoding 071000, Hebei, China

**Keywords:** Cervical cancer, Synchronous radiotherapy, Recombinant human Rh-endostatinstatin

## Abstract

**Objective:**

Retrospective study and analysis of the safety and efficacy of Rh-Rh-endostatinstatin combined with simultaneous radiotherapy in the treatment of cervical cancer.

**Methods:**

A retrospective study was used to enroll cervical cancer patients who received Rh-endostatin combined with simultaneous radiotherapy (observation group) or radiotherapy alone (control group) from January 2019 to December 2022 in the Affiliated Hospital of Hebei University, and RECIST 1.1 criteria were used to evaluate the recent efficacy, and the WHO Adverse Reaction Scale for Anti-cancer Drugs to evaluate the toxic and side effects.

**Results:**

The difference between PR, SD, PD, ORR and DCR of the two groups was not statistically significant(*P*>0.05), and the CR of the observation group was significantly higher than that of the control group(*P*<0.05). The proportion of neutropenia, hypertension, arrhythmia, hemoglobin reduction in the observation group was significantly higher than that in the control group, and the proportion of nausea and vomiting was significantly lower than that in the control group(*P*<0.05), and there was no significant difference in other adverse reactions(*P*>0.05). After intervention, the CD3+, CD3-CD19+, CD16+CD56+, CEA, CY211 of both groups were significantly lower than before treatment(*P*<0.05). After treatment, CD3+, CD3-CD19+, CD16+CD56+ were significantly higher in the observation group than in the control group, and WBC and PLT were significantly lower than before treatment(*P*<0.05). The HPV conversion rate of the observation group was significantly higher than that of the control group(*P*<0.05).

**Conclusions:**

Our finding revealed that Rh-endostatinstatin combined with simultaneous radiotherapy showed better clinical outcomes and favorable toxic profile than that of radiotherapy alone in the treatment of cervical cancer.

## INTRODUCTION

Cervical cancer is a common type of malignant tumor disease in women and ranks among the top four cancer incidences in women in terms of morbidity and mortality.^1^ Cervical cancer is a high risk factor for women’s health and safety.^2^

In the late stages, radiotherapy interventions for recurrent cervical cancer still have poor intervention effects. However, due to the hotspots of local invasion and metastasis, the overall survival rate of intermediate and late-stage recurrent cervical cancer has not yet exceeded 50%^3^, and the invasive and metastatic characteristics of malignant tumors cannot be controlled by radiotherapy alone. To enhance the effectiveness of interventions for intermediate and advanced malignancies, it is necessary to reduce local tumor load, inhibit metastatic characteristics, and eliminate subclinical lesions.^4^

Blood metastasis is the main mode of tumor metastasis and the only channel for tumor tissue to obtain nutrients. Inhibiting the proliferation and migration of vascular Rh-endostatinthelial cells can control tumor spread and metastasis.^5^,^6^ Rh-endostatinthelial inhibitors are currently the most effective vasopressors and can inhibit a variety of tumors.^7^ Rh-endostatin is a vascular Rh-endostatinthelial inhibitor drug successfully developed in China, which is a national class I new drug.^8^ A series of studies have shown that Rh-endostatin can inhibit angiogenesis and block the proliferation and metastasis of tumors.^9^-^11^ The efficacy and tolerability of Rh-endostatin combined with simultaneous radiotherapy as a first-line treatment for malignant tumors has been recognized in the clinical field.^12^-^16^

## METHODS

This was a retrospective study. A total of 80 patients with cervical cancer admitted in the Oncology Department of The Affiliated Hospital of Hebei University from January 2019 to December 2022 were enrolled, and they were divided into an observation group and a control group according to the intervention protocol they received during the consultation ( with the control group being radiotherapy cases alone and the observation group being Rh-endostatin combined with simultaneous radiotherapy cases, with 40 cases of cervical cancer in each group.)

### Ethical Approval:

This study was approved by The Affiliated Hospital of Hebei University Cancer Hospital ethics committee (No.: HDFYLL-KY-2023-063; date: April 25, 2023). As this was a retrospective study, the individual informed consent was waived.

### Inclusion criteria:

- Aged from 18 to 75 years old.
- Pathologically confirmed diagnosis of cervical cancer (inoperable cervical squamous cell carcinoma).
- No other treatment prior to this visit.
- FIGO stage IIb-IVa.
- KPS score ≥ 70 or ECOG (Eastern Cooperative Oncology Group) score 0-1.
- No contraindication to radiotherapy.
- Informed and agreed to participate in the study.
- At least six months of expected survival time.

### Exclusion criteria:

- Previous antineoplastic treatment.
- Cases of chronic functional disease.
- Located in pregnancy and lactation.
- Cases of other malignancies.

### Radiotherapy:

Patients in both groups were treated with 6-MV X-ray external radiation combined with 192Ir high-dose-rate intracavitary after loading. Synchronous chemotherapy: In the control group, the TP regimen was started from the first day of radiotherapy, paclitaxel was combined with platinum drugs, and the specific dosing regimen was as follows: cisplatin 25 mg/m^2^, intravenous infusion for 30-60 minutes; paclitaxel 40 mg/m^2^, intravenous infusion for more than 60 minutes;, once a week, three times a week, for 21 days.

### Anti-angiogenic treatment:

The observation group received Rh-endostatin intervention based on the treatment regimen of the control group, and the specific dosing regimen was as follows: Rh-endostatin 7.5 mg/m^2^/day for seven days from day 1-14 for one treatment cycle, and a total of two treatment cycles were carried out.

### Follow-up time:

All patients were followed up for the first time at the end of treatment, then every three months, and every six months after two years, with a final follow-up in December 2022.

### Efficacy evaluation and assessment:

RECIST1.1 criteria were used to evaluate the recent efficacy, and the observed indexes were complete remission (CR), partial remission (PR), disease progression (PD), and disease stability (SD). Objective remission rate (ORR) = (CR+PR)/total number of cases × 100%, disease control rate (DCR) = (CR+PR+SD)/total number of cases × 100%. PFS/OS/DMFS/LRRFS. The WHO anticancer drug adverse reaction scale was used to evaluate the toxic and side effects, which was divided into 0-4 grades. The hematological observation indexes of the two groups were tested before and after the intervention, including the clinical routine indexes and tumor indexes, and the differences in their intra-group levels before and after the treatment as well as the changes between groups were compared.

### Statistical analysis:

SPSS 26.0 (IBM SPSS Statistics 26.0) was used for the statistical analysis of the data.

## RESULTS

The comparison of general information of patients in both groups is shown in Table-I. In short, this indicated that the baseline information of the two groups of patients was comparable(P<0.05). The recent efficacy comparison between the two groups is shown in Table-II. The results of the χ2 test showed that the differences in PR, SD, PD, ORR, and DCR between the two groups were not statistically significant (p>0.05), while the CR in the observation group was significantly higher than that in the control group, and the difference was statistically significant (p<0.05).

**Table-I:** Comparison of general information between two groups of patients

Clinical features	Observation group(n=40)	Control group (n=40)	t/Z/χ^2^	p
Age	59.13±9.85	58.63±14.98	0.176	0.860
BMI	24.95±2.31	25.16±2.91	0.344	0.732
KPS			0.503	0.478
80	15(37.5)	12(30.0)		
90	25(62.5)	28(70.0)		
Menopause			0.549	0.459
After	30(75.0)	27(67.5)		
Before	10(25.0)	13(32.5)		
Hypertension			0.474	0.491
Yes	14(35.0)	17(42.5)		
No	26(65.0)	23(57.5)		
Diabetes			0.287	0.592
Yes	8(20.0)	10(25.0)		
No	32(80.0)	30(75.0)		
HPV-positive			0.263	0.608*
No	3(7.5)	1(2.5)		
Yes	37(92.5)	39(97.5)		
FIGO stage			-0.832	0.406
IIB	2(5.0)	3(7.5)		
IIIA	7(17.5)	10(25.0)		
IIIB	9(22.5)	8(20.0)		
IIIC	15(37.5)	13(32.5)		
IVA	7(17.5)	6(15.0)		
Degree of divergence			-1.052	0.293
Low	17(42.5)	22(55.0)		
Mid	14(35.0)	11(27.5)		
High	9(22.5)	7(17.5)		
Tumor size			2.464	0.116
<4cm	15(37.5)	22(55.0)		
≥4cm	25(62.5)	18(45.0)		
Histological type				
Squamous				
Adenocarcinoma				

**Table-II:** Comparison of short-term efficacy between the two groups [n(%)]

Efficacy	Control group(n=40)	Observation group(n=40)	χ^2^	P
CR, N (%)	24(60.0)	33(82.5)	4.943	0.026
PR, N (%)	10(25.0)	4(10.0)	3.117	0.077
SD, N (%)	4(10.0)	2(5.0)	0.180	0.671*
PD, N (%)	2(5.0)	1(2.5)	0.000	1.000*
ORR, N (%)	34(85.0)	37(92.5)	0.501	0.479*
DCR, N (%)	38(95.0)	39(97.5)	0.000	1.000*

***Note:*** ORR=CR+PR; DCR=CR+PR+SD.

* Fisher’s exact probability method was used.

The χ2 test showed that the difference in the number of patients with leukopenia, thrombocytopenia, hematuria, proteinuria, infection, diarrhea, reduced ejection fraction, ALT/AST, fatigue, radiation cystitis, genitourinary system reaction, radiation proctitis, and radiation skin damage was not statistically significant (p>0.05), while the proportion of neutropenia, hypertension, arrhythmia and hemoglobin reduction in the observation group was significantly higher than that in the control group, and the proportion of nausea and vomiting was significantly lower than that in the control group, and the differences were statistically significant (p<0.05).Table-III.

**Table-III:** Comparison of adverse reactions during treatment between two groups of patients.

Adverse reaction	Grouping	Adverse reaction grading					No. of patients (%)	χ^2^	p

		0	1	2	3	4			
Leucopenia	Observation group	17	14	9	0	0	23(57.5)	0.051	0.822
	Control group	18	10	10	2	0	22(55.0)		
Neutropenia	Observation group	15	20	3	2	0	25(62.5)	4.053	0.044
	Control group	24	13	2	1	0	16(40.0)		
Thrombocytopenia	Observation group	33	6	1	0	0	7(17.5)	0.313	0.576
	Control group	31	9	0	0	0	9(22.5)		
Hematuria	Observation group	32	8	0	0	0	8(20.0)	0.287	0.592
	Control group	30	9	1	0	0	10(25.0)		
Proteinuria	Observation group	35	5	0	0	0	5(12.5)	0.000	1.000*
	Control group	36	4	0	0	0	4(10.0)		
Hypertension	Observation group	31	9	0	0	0	9(22.5)	5.165	0.023
	Control group	38	2	0	0	0	2(5.0)		
Infection	Observation group	33	5	2	0	0	7(17.5)	0.672	0.412
	Control group	30	6	4	0	0	10(25.0)		
Nausea	Observation group	23	9	6	2	0	17(42.5)	6.146	0.013
	Control group	12	13	11	4	0	28(70.0)		
vomiting	Observation group	29	11	0	0	0	11(27.5)	7.366	0.007
	Control group	17	20	3	0	0	23(57.5)		
Diarrhea	Observation group	34	4	2	0	0	6(15.0)	0.457	0.499
	Control group	36	3	1	0	0	4(10.0)		
Decreased ejection fraction	Observation group	35	5	0	0	0	5(12.5)	0.392	0.531
	Control group	33	7	0	0	0	7(17.5)		
Arrhythmia	Observation group	32	8	0	0	0	8(20.0)	4.507	0.034*
	Control group	39	1	0	0	0	1(2.5)		
ALT/AST	Observation group	34	5	1	0	0	6(15.0)	0.738	0.390
	Control group	31	6	3	0	0	9(22.5)		
Fatigue	Observation group	16	13	11	0	0	24(60.0)	0.487	0.485
	Control group	13	13	14	0	0	27(67.5)		
Radiocystitis	Observation group	38	2	0	0	0	2(5.0)	0.180	0.671*
	Control group	36	4	0	0	0	4(10.0)		
Genitourinary reactions	Observation group	37	3	0	0	0	3(7.5)	0.263	0.608*
	Control group	39	1	0	0	0	1(2.5)		
Radioactive proctitis	Observation group	40	0	0	0	0	0(0.0)	0.000	1.000*
	Control group	39	1	0	0	0	1(2.5)		
Radiation skin lesion	Observation group	38	2	0	0	0	2(5.0)	0.180	0.671*
	Control group	36	4	0	0	0	4(10.0)		
Reduced hemoglobin	Observation group	17	17	5	1	0	23(57.5)	4.073	0.044
	Control group	26	11	3	0	0	14(35.0)		

***Note:***

* Corrected χ2 test was used.

The comparison of the observed indexes before and after treatment between the two groups is shown in Table-IV. The results of CD3+, CD3-CD19+, CD16+CD56+, CEA, and CY211 before and after treatment in the two groups by Mann-Whitney U test showed that the differences in CD3+, CD3-CD19+, CD16+CD56+, CEA, CY211 before treatment in the two groups and CEA, CY211 after treatment were not statistically significant (p>0.05), while CD3+, CD3-CD19+, CD16+CD56+ were significantly higher in the observation group than in the control group after treatment, and all differences were statistically significant (p<0.05).

**Table-IV:** Comparison of observation indexes before and after treatment between two groups of patients.

Index	Time Point	Observation group(n=40)	Control group(n=40)	t/Z	P
CD3+	Pre-treatment	906.00(680.50,1348.50)	1003.00(326.75,1782.50)	-0.255	0.799
	Post-treatment	450.00(324.00,733.25)	290.00(140.75,577.50)	-2.954	0.003
	Z	-4.247	-4.785	-	-
	P	<0.001	<0.001	-	-
CD3-CD19+	Pre-treatment	157.00(91.25,236.75)	138.00(41.25,244.25)	-0.544	0.587
	Post-treatment	98.50(40.75,208.50)	53.00(28.25,78.75)	-2.704	0.007
	Z	-3.357	-4.221	-	-
	P	0.001	<0.001	-	-
CD16+CD56+	Pre-treatment	185.00(110.00,303.00)	181.50(108.25,312.25)	-0.159	0.874
	Post-treatment	98.00(74.25,144.75)	66.50(42.25,140.00)	-2.242	0.025
	Z	-3.784	-5.041	-	-
	P	<0.001	<0.001	-	-
WBC	Pre-treatment	6.57±1.99	6.86±2.18	0.620	0.537
	Post-treatment	6.27±1.70	5.68±1.32	1.736	0.087
	t	0.679	4.802	-	-
	P	0.501	<0.001	-	-
HGB	Pre-treatment	118.20±19.66	117.23±24.17	0.198	0.844
	Post-treatment	115.05±18.64	114.58±22.41	0.103	0.918
	t	1.230	0.458	-	-
	P	0.226	0.650	-	-
PLT	Pre-treatment	293.15±82.85	299.85±103.23	0.320	0.750
	Post-treatment	284.70±60.28	275.05±90.58	0.561	0.577
	t	0.589	3.683	-	-
	P	0.559	0.001	-	-
CEA	Pre-treatment	2.14(1.57,6.12)	4.15(1.51,6.72)	-0.698	0.485
	Post-treatment	1.44(1.17,2.33)	1.83(1.19,3.02)	-0.914	0.361
	Z	-4.073	-4.577	-	-
	P	<0.001	<0.001	-	-
CY211	Pre-treatment	3.25(1.99,4.26)	3.16(2.27,5.10)	-0.371	0.711
	Post-treatment	1.63(1.32,2.20)	1.89(1.11,2.89)	-0.346	0.729
	Z	-5.000	-4.442	-	-
	P	<0.001	<0.001	-	-

The results of CD3+, CD3-CD19+, CD16+CD56+, CEA, and CY211 before and after treatment within the group showed by Wilcoxon test that CD3+, CD3-CD19+, CD16+CD56+, CEA, and CY211 were significantly lower than those before treatment in both groups, and the differences were all statistically significant (p<0.05). Similarly, the results of WBC, HGB, and PLT before and after treatment in both groups by independent sample t-test showed that the differences between WBC, HGB, and PLT before as well as after treatment in both groups were not statistically significant (p>0.05).

The results of WBC, HGB, and PLT before and after treatment within the group by paired sample t-test showed that the differences in HGB before and after treatment as well as WBC and PLT before and after treatment in the observation group were not statistically significant in both groups (p>0.05), while WBC and PLT after treatment in the control group were significantly lower than those before treatment, and the differences were statistically significant (p<0.05).

Before treatment, there were 37 HPV-positive cases in the observation group and 39 HPV-positive cases in the control group, and after the intervention, 34 patients in the observation group turned negative for HPV, with a conversion rate of 91.9% (34/37), and a total of 28 patients in the control group turned negative for HPV, with a conversion rate of 71.8% (28/39), and the HPV conversion rate of patients in both groups was significantly higher in the observation group than in the control group. The difference was statistically significant (χ2=5.103, p<0.05).

## DISCUSSION

In terms of adverse effects, the incidence of neutropenia, hypertension, arrhythmias, and decreased hemoglobin during treatment was relatively higher in the observation group cases, and the incidence of nausea and vomiting was relatively lower. Neutropenia is one of the common adverse effects of Rh-endostatin treatment, which may lead to neutropenia due to the effect of Rh-endostatin on bone marrow hematopoietic function. Hypertension and cardiac arrhythmias are also common adverse effects of Rh-endostatin because of the effects of the drug components on the cardiovascular system. In addition, the hematopoietic system of the body is affected during the application of Rh-endostatin, and the hemoglobin index of the tutor is reduced. In terms of other adverse reactions, no significant abnormalities were found in this study, and the main adverse reactions were concentrated in grades 0-2, which were controllable adverse reactions, so it can be tentatively inferred that the safety of Rh-endostatin combined with simultaneous radiotherapy intervention is high.

Cervical cancer is an important cancer affecting women’s reproductive health, and radiation therapy is the main treatment option for cervical cancer^17^. Existing studies show that the five-year survival rate of cervical cancer patients is at a low level, with only 34.3 % of cervical cancer patients in China^18^, so the medical field continues to search for more efficient and safe treatment options.

The relationship between malignant tumor growth and metastasis and neovascularization has been confirmed in the field of research, and the formation of neovascularization is a necessary condition, an important channel, and an absolute hub for the growth and metastasis of tumor cells.^19^ Therefore, anti-angiogenic therapy can effectively enhance the clinical treatment effect of malignant tumors. Vascular endothelial growth factor signalling is an effective target for cancer treatment, and tumours growth can be effectively inhibited by anti-angiogenic therapy without obvious side effects.^20^ In the treatment of cervical cancer, the use of oral anti-angiogenic drugs in the treatment of cervical cancer can extend the treatment time to 540 days, and the complication rate in the treatment is low, which has a certain degree of safety.^21^

Rh-endostatin is a recombinant human vascular Rh-endostatinthelial inhibitor drug developed in China, which can inhibit angiogenesis through pan-targeting, and numerous studies have confirmed that Rh-endostatin can inhibit the growth of tumor cells and enhance the effectiveness of malignant tumor intervention.^22^-^24^ Therefore, the present study was a retrospective analysis of the interventional utility of Rh-endostatin combined with concurrent radiotherapy based on the cervical cancer population.

In terms of recent efficacy, we can find that Rh-endostatin combined with synchronous radiotherapy can effectively increase the complete remission rate of cervical cancer, and compared with synchronous radiotherapy intervention alone, Rh-endostatin combined with synchronous chemotherapy can induce more cervical cancer patients to achieve complete remission, and Rh-endostatin can inhibit angiogenesis, blocking the supply of nutrients and nutrients to tumor cells, and achieve the purpose of killing tumor cells. The complete remission rate of the observation group was higher because Rh-endostatin could inhibit angiogenesis, blocking the supply of nutrients and nutrients to tumor cells, and kill tumor cells. The objective remission rate and disease control rate of the two groups did not show any significant difference, which may be related to the limited sample size.

In terms of serologically related indexes, the levels of CD3+, CD3-CD19+, and CD16+CD56+ indexes were higher in the observation group cases than in the control group after treatment. CD3+, CD3-CD19+, and CD16+CD56+ are important indexes for the assessment of the body’s immune function, and Rh-endostatin has a strong modulating effect on immune function, therefore, compared with the single radiotherapy regimen, Rh-endostatin the assessment results of CD3+, CD3-CD19+, CD16+CD56+ index levels in cervical cancer cases were more excellent after combined with simultaneous radiotherapy intervention.

In terms of HPV infection rate, after treatment, the HPV conversion rate of cases in the observation group was significantly better than that of the control group. Cervical cancer is closely related to HPV infection, and Rh-endostatin can have an inhibitory effect on the DNA synthesis of HPV, reducing the replication as well as the production of the virus, thus achieving an enhanced HPV conversion rate.

### Limitations:

It includes small sample size, small course of treatment, and short follow-up time. In the follow-up study, we will further clarify the application of Rh-endostatin, confirm the clinical value of Rh-endostatin through more basic research evidence, provide a reference basis for the further application of Rh-endostatin, and also provide a new direction for the treatment of cervical cancer and other malignant tumor diseases.

## CONCLUSIONS

This study further confirmed the safety and efficacy of Rh-endostatin in combination with concurrent radiotherapy in the treatment of cervical cancer and also validated the effectiveness of Rh-endostatin in intervening in HPV infection.

### Authors’ Contributions:

**XZ and QL** carried out the studies, participated in collecting data, drafted the manuscript, are responsible and accountable for the accuracy or integrity of the work.

**KL** performed the statistical analysis and participated in its design.

**HS** participated in acquisition, analysis, or interpretation of data and drafted the manuscript.

All authors read and approved the final manuscript.
